# Evaluation of continuous constant current and continuous pulsed current in sweat induction for cystic fibrosis diagnosis

**DOI:** 10.1186/s12890-018-0696-3

**Published:** 2018-09-14

**Authors:** Carla Cristina Souza Gomez, Fernando Augusto Lima Marson, Maria Fátima Servidoni, Antônio Fernando Ribeiro, Maria Ângela Gonçalves Oliveira Ribeiro, Veruska Acioli Lopes Gama, Eduardo Tavares Costa, José Dirceu Ribeiro, Francisco Ubaldo Vieira Junior

**Affiliations:** 1 Department of Pediatrics, School of Medical Sciences, University of Campinas, Cidade Universitária Zeferino Vaz, Barão Geraldo, Campinas, São Paulo 13083-887 Brazil; 2 Center for Research in Pediatrics, School of Medical Sciences, University of Campinas, Cidade Universitária Zeferino Vaz, Barão Geraldo, Campinas, São Paulo 13083-887 Brazil; 3 Department of Medical Genetics, School of Medical Sciences, University of Campinas, Cidade Universitária Zeferino Vaz, Barão Geraldo, Campinas, São Paulo 13083-887 Brazil; 4 Gastrocentro – Endoscopy Unit, University of Campinas, Cidade Universitária Zeferino Vaz, Barão Geraldo, Campinas, São Paulo 13083-872 Brazil; 5 Center for Biomedical Engineering, University of Campinas, Cidade Universitária Zeferino Vaz, Barão Geraldo, Campinas, São Paulo 13083-970 Brazil; 6 Department of Biomedical Engineering, Faculty of Electrical and Computer Engineering, University of Campinas, Cidade Universitária Zeferino Vaz, Barão Geraldo, Campinas, São Paulo 13083-881 Brazil; 7 Federal Institute of Education, Science and Technology of Sao Paulo, Campus Campinas, km 143.5, Campinas, São Paulo 13069-901 Brazil

**Keywords:** Continuous constant current, Continuous pulsed current, Sweat test, Sinusoidal pulsed current, Triangular pulsed current

## Abstract

**Background:**

The sweat test (ST) is the gold standard for the diagnosis of cystic fibrosis (CF). However, little is known about sweat induction using different types of currents and waves. In this context, our objective was to develop a device to induce sweat and compare the use of continuous constant current (CCC) and continuous pulsed current (CPC) in individuals with CF and healthy controls.

**Methods:**

A prospective cross-sectional study with experimental intervention. The variables of gender, ethnicity, age, and body mass index (BMI) were considered. The method of Gibson and Cooke was used, and the following markers were evaluated: sweat weight, electrical impedance, sufficient sweat amount, and CF diagnosis. Triangular (TPC) or sinusoidal (SPC) pulsed current was applied to the right arm, and CCC was applied to the left arm.

**Results:**

The study analyzed 260 individuals, 141/213 (54.2%) were female participants, 135/260 (51.9%) were Caucasians. The distribution of individuals by concentration of chloride at the ST was: (CF) 26/260 (10%); (borderlines) 109/260 (41.9%); (healthy) 97/260 (37.3%); (insufficient weight in sweat) 28/260 (10.8%). No association was observed between the sufficient sweat amount to perform the ST when we compared the currents. However, the SPC showed a higher amount of sweat weight. Using Bland and Altman plot considering the agreement between the sweat chloride values achieved from CPC [SPC and TPC] and CCC, there was no proportional bias and mean values are unrelated and only explain less than 8% of the variation. Moreover, TPC presented higher electrical impedance when compared with SPC and CCC. SPC presented lower electrical impedance and higher sweat weight than CCC. Male participants presented lower electrical impedance and higher sweat weight with CCC and TPC, and higher sweat weight with SPC.

**Conclusions:**

The evaluated currents are safe and able to induce and produce sweat in sufficient quantities for the ST. SPC presented lower electrical impedance when compared with other currents. The use of SPC is recommended to induce sweat in patients with sweat problems. Finally, ethnicity, gender, age and BMI did not influence sweat induction at the ST, and no side effect was observed in our study.

**Electronic supplementary material:**

The online version of this article (10.1186/s12890-018-0696-3) contains supplementary material, which is available to authorized users.

## Background

The evaluation of the cystic fibrosis transmembrane conductance regulator (CFTR) function through the sweat test was a milestone for the diagnosis of cystic fibrosis (CF) (OMIM: #219700). The sweat test was created around six decades ago by Gibson and Cooke (1959), and so far, it has been considered the main tool for the diagnosis of CF [1]. An early diagnosis due to the sweat test ensured advances in lowering the deterioration of nutritional status and lung function. In addition, the sweat test enabled a better understanding of the disease and the evaluation of the efficacy of new drugs by personalized/precision medicine [2–5].

The sweat test uses the pilocarpine iontophoresis method to induce sweat and evaluate the amount of chloride in sweat. The diagnosis of CF is confirmed when the levels of chloride in sweat are equal to or greater than 60 mmol/L considering two sweat tests performed at different moments [1, 2, 6–8]. Although the sweat test is the gold standard for the diagnosis of CF, with numerous published guidelines that recommend it, the literature reports challenges when conducting this test [9–17].

The sweat test involves three stages: induction, collection and measurement of electrolytes. Efficacy is related to the experience of the professional who conducts the test and the use of appropriate equipment to induce sweat. Due to the sweat test complexity, many laboratories worldwide have reported several challenges when conducting it. Then, to standardize the sweat test, the United States of America and Europe were the pioneers in the development of guidelines and reports about the test [7–18]. Brazil does not have its own guideline and several methods are used when conducting the sweat test.

The challenges to conduct the sweat test start in sweat induction. The equipment for sweat induction used in Brazil is mostly manufactured by the CF university centers and does not have authorization from the national health surveillance agency, except for Macroduct^®^ (Wescor^®^, Utah, USA), a system that has been used by some centers [19, 20].

In the literature, commonly, iontophoresis devices to induce sweat use the constant continuous current of one ampere [21–23]. However, the literature also has reports of efficient delivery of substances through the skin with the use of continuous pulsed current, without risks of burning and discomfort when compared with continuous constant current, a fact that is still controversial in CF [21, 22].

This study aimed to develop a low cost sweat induction device and compare the volume of sweat obtained using continuous constant current and continuous pulsed current in individuals with CF and healthy controls of all age groups and both genders.

## Methods

### Individuals enrolled in the study

We performed a prospective cross-sectional study with experimental intervention, unblinded and nonrandomized, involving individuals with and without CF of all age groups, of both genders, Caucasians or non-Caucasians. Individuals with CF were recruited from the CF Reference Center of the University of Campinas. Nursery children, adult staff and university students comprised the remaining sample as volunteers.

The individuals with CF were diagnosed through: compatible clinical history, two chlorides quantifications in sweat with values greater than 60 mmol/L, and/or a genetic study of confirmed *CFTR* gene mutations (OMIM: # 602421). The volunteers did not have any known chronic disease.

The project was approved by the Research Ethics Committee of the University of Campinas (#809.090/2014). Written informed consent for participation in the study was obtained from participants or, where participants are children, from a parent or guardian.

### Devices used to induce sweat

The sweat-inducing device used in the sweat test was developed by the Instituto Federal de São Paulo and the Biomedical Engineering Center of the University of Campinas. The iontophoresis device used is portable and easy to use, and it offers the possibility to select between continuous pulsed current and continuous constant current, with triangular pulsed current, sinusoidal pulsed current and continuous constant current waveform, at the frequency of 1000 Hz, maximum output current of one mA, and embedded software to support the settings for data control and acquisition, with two brass electrodes of 30 mm diameter.

The device was developed from a microcontroller and a circuit that generates the continuous constant current and continuous pulsed current signals. The effective root mean square (RMS) value of the current was calculated from the numerical integration of the sampled currents.

During the sweat test, the applied voltage and current values were stored in a memory card every 0.8 s in 32-slot sampling blocks.

From the voltage and current values recorded in the memory card, curves were reconstructed relative to the waveforms (voltage and current) of each test. With the help of Matlab^®^ software, mean electrical impedance was calculated for the total time of the test (10 min). The stimulation of 10 min was different from Gibson and Cooke method [1] regarding two main factors: (i) there was evaluated an amperage of one mA to induce the sweating; (ii) in a previous study, the time of 10 min presented a greater sweating in the sweat test [23].

To ensure the safety and feasibility for the human individuals enrolled in our study, the sweats tests were performed at the tertiary hospital in the presence of a medical doctor in a reference laboratory that performed the sweats tests in our institution during the last 30 years.

### Clinical markers evaluated

The variables of gender (male/female), self-declared ethnicity, age (years), and body mass index were considered. The body mass index was calculated using the following formula: weight (Kg)/height^2^ (m), and the z-score analysis for age was included, with data categorized as accentuated slenderness, slenderness, eutrophic, overweight, obesity, and severe obesity. The clinical markers were compared with the results achieved in the sweat tests.

### Sweat test

The method of Gibson and Cooke was performed in the two stages of the sweat test: induction and collection to analysis the concentration of chloride [1].

The distance of two cm and five cm between the electrodes were adopted for newborns and the other participants, respectively. To minimize the risk of burning, the gauze was kept completely moist with pilocarpine, and the electrode was attached onto the gauze with an elastic band to prevent electrode displacement on the arm.

For each patient, the mean electrical impedance was calculated for the electrode, gauze and skin assembly during the induction time by the Ohm’s law, using the following equation: [Z = V_RMS_ / I_RMS_ (Ω)]. Being: Z = composite impedance (Ω); V_RMS_ = effective voltage measured (volts root mean square); I_RMS_ = effective current measured (current root mean square). To collect sweat, a 17.5 cm^2^ filter paper covered with plastic and crepe bandage was used. The concentration of chloride was obtained by manual titration [24], also the analysis was done after an extensively trainee with a technician that performed the sweat test for 30 years using the same protocol. In this context, a gauze was soaked with pilocarpine only to stimulate sweating, and subsequently the sweat collection was performed with filter paper (Whatman™ 1001–125, Little Chaltfon, Buckinghamshire, UK) after cleansing the arm.

To minimize the bias, we performed the sweat test using a standard protocol. Also, negative and positive controls were quantified, at the same time, with the sample individuals. The reagents were strictly conditioned and we used: (i) Standard – Sodium chloride (100 mmol/L); (ii) Nitrate – mercury nitrate 1.1 mmol/L and 0.9 mmol/L nitric acid; (ii) Color reagent – Mercury thiocyanate two mmol/L, ferric nitrate 17 mmol/L and nitric acid 30 mmol/L. In our sample, we observed a higher variability in sweat test analysis in individuals with the lower concentration of chloride. The manual titration is dependent from the experience of who does the exam, but this technique is suitable to perform the sweat test, and the exam was done in supervision of a technique that performed the test during the last 30 years, as previously declared.

At the sweat test, 10 and 30 min were used for sweat induction and collection, respectively, as well as one mA current and 1000 Hz frequency to pulsed current.

With the sweat test, the following data were evaluated: sweat weight (mg), mean electrical impedance, sufficient amount of sweat obtained during induced sweat (weight greater than 75 mg), and diagnostic parameters of CF (healthy individuals = chloride < 30 mmol/L; borderline = chloride ≥ 30 mmol/L to < 60 mmol/L; CF = chloride ≥ 60 mmol/L) [8].

### Experiment description

**Fig. 1:**
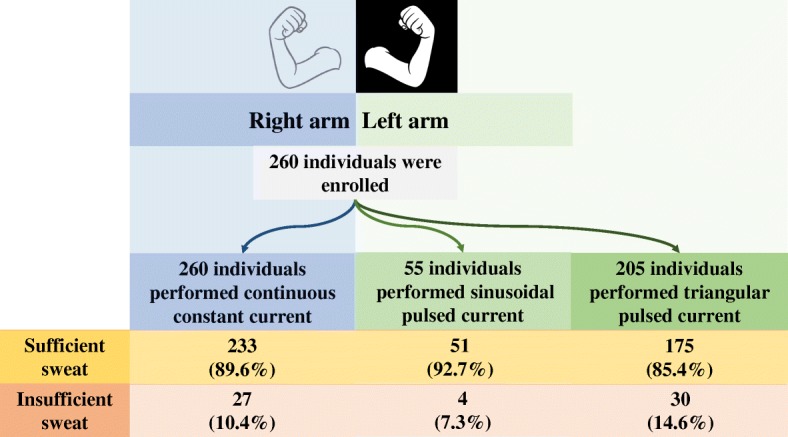
Study protocol and experimental description. The study enrolled a total of 260 individuals. All the 260 individuals performed the sweat test with continuous constant current at the right arm. Also, from 260 individuals, 205 performed the sweat test with triangular pulsed current and 55 with sinusoidal pulsed current at the left arm. The comparison between continuous constant current and continuous pulsed current was based on related samples. The comparison between sinusoidal constant current and triangular constant current was based on unrelated samples

**Fig. 2:**
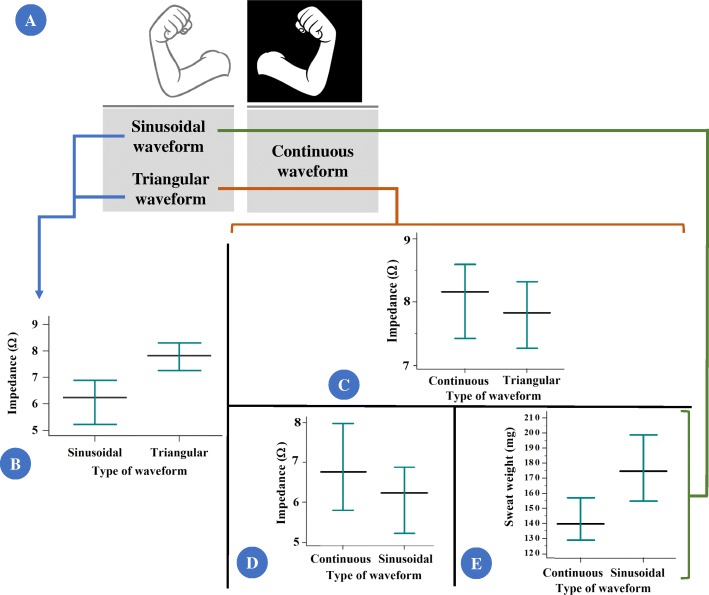
The comparison between continuous constant current (CCC) and sinusoidal pulsed current (SPC) and triangular pulsed current (TPC) for electrical impedance and sweat weight showed: (i) TPC presented higher electrical impedance values when compared with SPC and CCC; (ii) SPC presented lower electrical impedance and higher sweat weight when compared with CCC. **a** Arm site where induction was performed using CCC and continuous pulsed current. **b** Comparison between SPC and TPC for electrical impedance. (SPC) *N* = 54; 6.14 ± 2.08; 6.23 (2.12 to 11.05); 5.57 to 6.71; (TPC) *N* = 201; 7.94 ± 3.16; 7.82 (1.94 to 17.76); 7.5 to 8.38. **c** Comparison between CCC and TPC for electrical impedance. (CCC) *N* = 197; 8.9 ± 4.8; 8.15 (1.12 to 38.33); 8.23 to 9.60; (TPC) *N* = 197; 7.97 ± 3.2; 7.83 (1.94 to 17.76); 7.52 to 8.41. **d** Comparison between CCC and SPC for electrical impedance. (CCC) *N* = 54; 7.29 ± 2.98; 6.76 (2.95 to 17.11); 6.47 to 8.1; (SPC) *N* = 54; 6.14 ± 2.08; 6.23 (2.12 to 11.05); 5.57 to 6.71. **e** Comparison between CCC and SPC for sweat weight. (CCC) *N* = 55; 146 ± 46.1; 141 (50 to 234); 133.3 to 158.8; (SPC) *N* = 55; 179 ± 70.2; 176 (46 to 433); 159.7 to 197.1. All *p*-values were below 0.001. Data are presented as: number of individuals; mean ± standard deviation; median (minimum to maximum); confidence interval for the mean value. Statistical analysis conducted through Wilcoxon signed-rank test and Mann-Whitney U test of independent samples. Alpha = 0.05. The currents are shown as Ω using the following equation: [Z = V_RMS_ / I_RMS_ (Ω)]; Z = composite impedance (Ω); V_RMS_ = effective voltage measured (volts root mean square); I_RMS_ = effective current measured (current root mean square). Also, the sweat weight is shown as milligrams. Only the associations with *p*-value < 0.05 was presented as figure

Triangular pulsed current or sinusoidal pulsed current was applied to the left arm of the participants, and continuous constant current to the right arm. The complete details can be visualized at Figs. 1 and 2a.

### Statistical analysis

In the statistical analysis, the sweat weight and electrical impedance of the electrode, gauze and skin assembly obtained with the application of continuous constant current, triangular pulsed current, and sinusoidal pulsed current were compared. In addition, the sweat weight and mean electrical impedance were evaluated in relation to the variables of ethnicity, age, gender and body mass index.

Statistical Package for the Social Sciences*,* version 23.0 (IBM^®^, Armonk, NY, USA), was used in data analysis. The charts were built with MedCalc 13.2.2 (MedCalc Software, Acacialaan 22, B-8400, Ostend, Belgium).

The numerical variables are presented in the study by measures of position and dispersion, and the categorical variables by absolute number and percentage.

In the comparative analysis between the data from the same individual, considering the different tests performed (continuous pulsed current versus continuous constant current), the Wilcoxon signed-rank test of related samples was applied. The Mann-Whitney U test of independent samples was applied to the variables compared between different individuals (triangular pulsed current versus sinusoidal pulsed current and clinical markers for ethnicity, age, gender and body mass index for sweat weight and electrical impedance). Also, we performed the association between the difference of continuous pulsed current and continuous constant current, regarding sweat weight of sinusoidal pulsed current and triangular pulsed current by Mannn-Whitney U test of independent samples. The comparison was conducted for sweat weight (whether or not sufficient for the quantification of the concentration of chloride) and CF diagnosis, in the comparison between right and left arms, using McNemar’s tests and the Wilcoxon signed-rank test (ordinal data), respectively. The comparison of z-score of body mass index with gender and ethnicity was performed using the χ^2^ test. The odds ratio and the 95% confidence interval were calculated for *p*-values below 0.05. Spearman’s correlation between age, body mass index, electrode, gauze and skin electrical impedance and concentration of chloride were performed considering the CF diagnosis with continuous constant current. Also, the Spearman’s correlation was used to compare the association between sweat chloride values and sweat weight (only sweat samples with sweat weight ≥ 75 mg were analyzed) regarding all the currents analyzed.

Moreover, a Bland and Altman plot were done to represent the sweat test diagnosis difference (continuous pulsed current – continuous constant current) (y axis) and mean sweat test values between tests current [(continuous pulsed current + continuous constant current) / 2] (x axis). Also, we calculate the linear regression coefficient between sweat test diagnosis difference and mean sweat test values.

The level of significance adopted in all analyses was α = 0.05.

## Results

The study analyzed 260 individuals, 141/260 (54.2%) were female participants, 135/260 (51.9%) were Caucasians, the body mass index was 22.42 ± 5.99 Kg/m^2^. The median age was 26 years, ranging from 0.1 to 77 years. The distribution of individuals by concentration of chloride at the sweat test was: (CF) 26/260 (10%); (borderline) 109/260 (41.9%); (healthy) 97/260 (37.3%); (insufficient weight in sweat - below 75 mg) 28/260 (10.8%) (Fig. 1).

In the study, a correlation was observed between the level of chloride and age (Spearman’s Rho = 0.178; *p*-value = 0.007) and body mass index (Spearman’s Rho = 0.163; *p*-value = 0.014). However, when the correlation between the level of chloride and age and body mass index was analyzed for the different CF diagnostic groups, no significant correlation was observed (*p*-value > 0.05). The correlation between electrode, gauze and skin assembly electrical impedance and age was positive with continuous constant current (Spearman’s Rho = 0.262; *p*-value < 0.001); triangular pulsed current (Spearman’s Rho = 0.256, *p*-value < 0.001); and sinusoidal pulsed current (Spearman’s Rho = 0.292, *p*-value = 0.032).

### Comparison between currents (Additional file 1)

(i) triangular pulsed current presented higher electrical impedance values when compared with sinusoidal pulsed current (Fig. 2b) (*p*-value < 0.001). However, no difference was observed in sweat weight between continuous pulsed current (*p*-value = 0.888);
(ii) triangular pulsed current presented higher electrical impedance values when compared with continuous constant current (*p*-value < 0.001) (Fig. 2c). However, no difference was observed in sweat weight considering the triangular pulsed current and continuous constant current (*p*-value = 0.188);
(iii) sinusoidal pulsed current presented lower electrical impedance (Fig. 2d) and higher sweat weight (Fig. 2e**)** when compared with continuous constant current (*p*-value < 0.001).

### Gender comparison for the evaluated markers (Additional file 2)

**Fig. 3:**
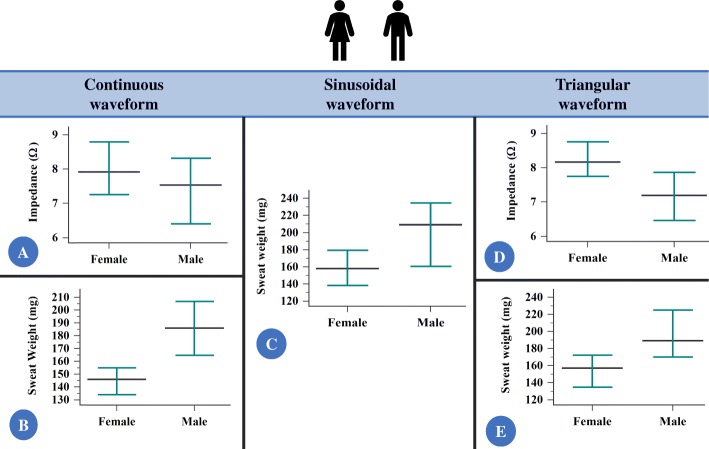
The comparison between female and male participants for the values of electrical impedance and sweat weight, according to the current applied showed: male presented lower electrical impedance [continuous constant current (CCC), triangular pulsed current (TPC)] and higher sweat weight [CCC, sinusoidal pulsed current (SPC), TPC] in relation to female. **a** Comparison for electrical impedance with CCC. (Female) *N* = 136; 9.3 ± 4.94; 7.91 (1.12 to 38.33); 8.46 to 10.14; (Male) *N* = 116; 7.67 ± 3.75; 7.53 (2.3 to 22.29); 6.98 to 8.36. **b** Comparison for sweat weight with CCC. (Female) *N* = 141; 155 ± 73; 146 (0 to 425); 142 to 167; (Male) *N* = 119; 193 ± 104; 186 (3 to 577); 174 to 211. **c** Comparison for sweat weight with SPC. (Female) *N* = 30; 153 ± 55; 158 (46 to 267); 132 to 174; (Male) *N* = 25; 208 ± 74; 209 (97 to 433); 177 to 238. **d** Comparison for electrical impedance with TPC. (Female) *N* = 109; 8.57 ± 3.17; 8.16 (2.33 to 17.76); 7.97 to 9.17; (Male) *N* = 92; 7.2 ± 2.98; 7.18 (1.94 to 16.33); 6.58 to 7.81. **e** Comparison for sweat weight with TPC. (Female) *N* = 109; 164 ± 88; 157 (6 to 535); 148 to 181; (Male) *N* = 92; 206 ± 115; 189 (9 to 699); 182 to 229. All *p*-values were below 0.003. Data are presented in legend as: number of individuals; mean ± standard deviation; median (minimum to maximum); confidence interval for the mean value; and in figure as median (black line) and 95% confidence interval (green line). Statistical analysis conducted through Wilcoxon signed-rank test and Mann-Whitney U test of independent samples. Alpha = 0.05. The currents are shown as Ω using the following equation: [Z = V_RMS_ / I_RMS_ (Ω)]; Z = composite impedance (Ω); V_RMS_ = effective voltage measured (volts root mean square); I_RMS_ = effective current measured (current root mean square). Also, the sweat weight is shown as milligrams. There is no impedance figure with sinusoidal pulsed current because the *p*-value > 0.05 was observed

(i) continuous constant current: male participants presented lower electrical impedance (*p*-value < 0.001; Fig. 3a) and higher sweat weight (*p*-value = 0.008; Fig. 3b) in relation to female participants. Body mass index in Kg/m^2^ was the same for both groups (*p*-value = 0.085); however, different values were obtained with the z-score analysis (*p*-value = 0.011) (Table 1);
(ii) sinusoidal pulsed current: male participants presented higher sweat weight (Fig. 3c) in relation to female participants (*p*-value = 0.007). However, no difference was observed in electrical impedance (*p*-value = 0.381) and body mass index (Kg/m^2^ – *p*-value = 0.287; z-score – *p*-value = 0.733) in gender comparison;
(iii) triangular pulsed current: male participants presented lower electrical impedance (*p*-value < 0.003; Fig. 3d) and higher sweat weight (*p*-value = 0.008; Fig. 3e) in relation to female participants. Body mass index in Kg/m^2^ was the same for both groups (*p*-value = 0.202); however, different values were obtained with the z-score analysis (*p*-value = 0.012) (Table 1).

**Table 1:** Comparison of body mass index adjusted by z-score of age for gender and ethnicity for continuous constant current and sinusoidal and triangular continuous pulsed current

Body mass index group		Gender			*p*-value
		Female	Male	Total	
Continuous constant current	Eutrophic	91	61	152	**0.011**
	Slenderness + accentuated slenderness^a^	7	18	25	
	Overweight + obesity + serious obesity^b^	42	39	81	
	Total	140	118	258	
Triangular continuous pulsed current	Eutrophic	78	52	130	**0.012**
	Slenderness + accentuated slenderness^c^	5	15	20	
	Overweight + obesity + serious obesity^d^	27	26	53	
	Total	110	93	203	
Body mass index group		Caucasians			p-value
		Yes	No	Total	
Continuous constant current	Eutrophic	68	84	152	**0.027**
	Slenderness + accentuated slenderness^e^	14	11	25	
	Overweight + obesity + serious obesity^f^	51	30	81	
	Total	133	125	258	
Triangular continuous pulsed current	Eutrophic	52	78	120	**0.007**
	Slenderness + accentuated slenderness^g^	12	8	20	
	Overweight + obesity + serious obesity^h^	34	19	53	
	Total	98	105	203	

Body mass index was adjusted by z-score (age); OR, odds ratio; 95%CI, confidence interval of 95%. The calculation of odds ratio used the degree of eutrophic as its parameter. Statistical analysis conducted by χ^2^ test. Alpha = 0.05. Statistically significant data are in bold

^a^OR = 0.261; 95%CI = 0.103 to 0.662

^b^OR = 0.722; 95%CI = 0.419 to 1.243

^c^OR = 0.222; 95%CI = 0.076 to 0.649

^d^OR = 0.692; 95%CI = 0.364 to 1.317

^e^OR = 1.572; 95%CI = 0.671 to 3.685

^f^OR = 2.1; 95%CI = 1.208 to 3.65

^g^OR = 2.25; 95%CI = 0.861 to 5.882

^h^OR = 2.684; 95%CI = 1.385 to 5.204

### Ethnicity comparison (Caucasians and non-Caucasians) for the evaluated markers (Additional file 3)

**Fig. 4:**
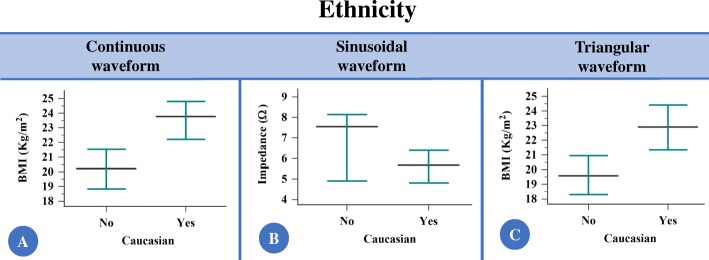
The comparison between Caucasians and non-Caucasians for the values of electrical impedance and body mass index, according to the current applied showed that the body mass index was higher in the group of Caucasians [continuous constant current (CCC) and triangular pulsed current (TPC)], and the sinusoidal pulsed current (SPC) was associated with lower electrical impedance in the group of Caucasians. **a** Body mass index with the application of CCC. (Non-Caucasians) *N* = 125; 21.26 ± 5.7; 20.21 (11.06 to 42.29); 20.25 to 22.27; (Caucasians) *N* = 133; 23.5 ± 6.07; 23.72 (0 to 38.2); 22.46 to 24.55; (*p*-value = 0.001). **b** Electrical impedance with the application of SPC. (Non-Caucasians) *N* = 20; 6.98 ± 2.36; 7.55 (3.19 to 11.05); 5.87 to 8.09; (Caucasians) *N* = 34; 5.64 ± 1.74; 5.66 (2.12 to 9.62); 5.03 to 6.25. (*p*-value = 0.037). **c** Electrical impedance with the application of TPC. (Non-Caucasians) *N* = 105; 20.6 ± 5.06; 19.57 (11.06 to 34.84); 19.61 to 21.58; (Caucasians) *N* = 98; 23.14 ± 6.19; 22.9 (0 to 38.2); 21.89 to 24.38. (*p*-value = 0.001). Data are presented in legend as: number of individuals; mean ± standard deviation; median (minimum to maximum); confidence interval for the mean value; confidence interval for the mean value; and in figure as median (black line) and 95% confidence interval (green line). Statistical analysis conducted through Wilcoxon signed-rank test and Mann-Whitney U test of independent samples. Alpha = 0.05. The currents are shown as Ω using the following equation: [Z = V_RMS_ / I_RMS_ (Ω)]; Z = composite impedance (Ω); V_RMS_ = effective voltage measured (volts root mean square); I_RMS_ = effective current measured (current root mean square). Also, the body mass index is shown as weight (Kg)/height^2^ (m). Only the associations with *p*-value < 0.05 was presented as figure

(i) continuous constant current: body mass index in Kg/m^2^ was higher in the group of Caucasians (*p*-value = 0.001), and different values were obtained for the groups with the z-score analysis (*p*-value = 0.027) (Fig. 4a and Table 1). However, no difference was observed in electrical impedance (*p*-value = 0.653) and sweat weight (*p*-value = 0.141);
(ii) sinusoidal pulsed current: electrical impedance was lower in the group of Caucasians (*p*-value = 0.037) (Fig. 4b). However, no difference was observed in sweat weight (*p*-value = 0.214) and body mass index (Kg/m^2^ – *p*-value = 0.861; z-score – *p*-value = 0.351);
(iii) triangular pulsed current: body mass index in Kg/m^2^ was higher in the group of Caucasians (*p*-value = 0.001), and different values were obtained for the groups with the z-score analysis (*p*-value = 0.007) (Fig. 4c and Table 1). However, no difference was observed in electrical impedance (*p*-value = 0.508) and sweat weight (*p*-value = 0.141).

### Comparison of number of exams with insufficient sweat weight and sweat test results between continuous constant current and sinusoidal and triangular continuous pulsed current

**Fig. 5:**
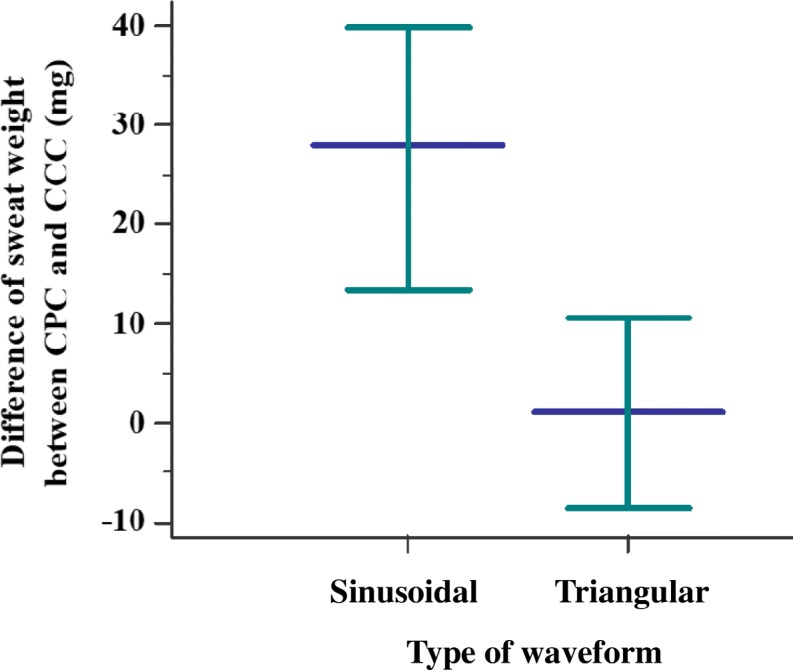
Difference between sweat weight achieved from continuous pulsed current (sinusoidal versus triangular) in relation to continuous constant current showed a higher amount of sweat weight in sinusoidal pulsed current. *p*-value = 0.02. (Sinusoidal pulsed current) *N* = 49; 34.63 ± 54.02; 28 (− 58 to 231); 19.12 to 50.15; (Triangular pulsed current) *N* = 180; − 0.96 ± 85.99; 1 (− 364 to 434); − 13.6 to 11.69. Data are presented in legend as: number of individuals; mean ± standard deviation; median (minimum to maximum); confidence interval for the mean value; and in figure as median (black line) and 95% confidence interval (green line). Statistical analysis conducted through Mann-Whitney U test of independent samples. Alpha = 0.05. The sweat weight is shown as milligrams

**Fig. 6:**
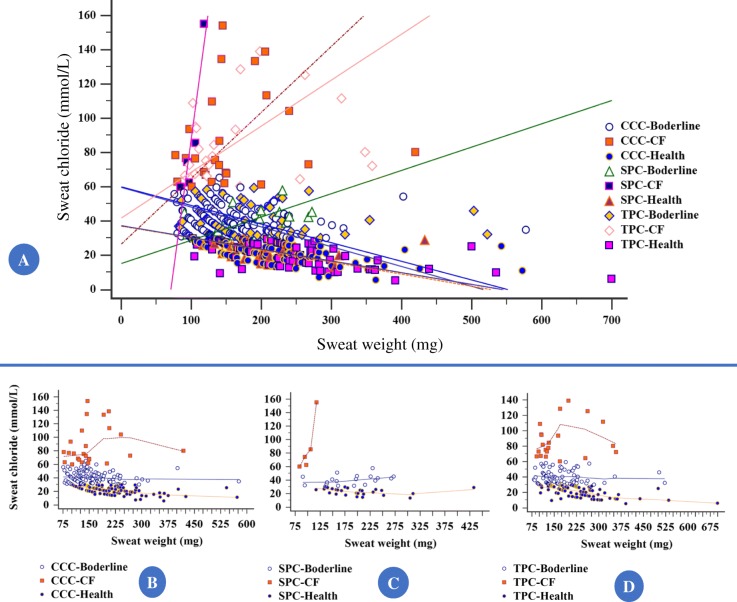
Spearman’s coefficient of Rank correlation (Rho) between chloride values achieved in the sweat test (mmol/L) and sweat weight (mg) showed there was a positive correlation between sweat chloride values and sweat weight. **a** All samples: Sample size = 460 (sweat weight below of 75 mg were excluded). Rho = − 0.482; 95% confidence interval for Rho = − 0.549 to − 0.409. *p*-value < 0.0001. CCC-CF = 26/460 (5.7%); CCC-borderline = 108/460 (23.5%); CCC-health = 97/460 (21.1%); SPC-CF = 5/460 (1.1%); SPC-borderline = 19/460 (4.1%); SPC-health = 28/460 (6.1%); TPC-CF = 21/460 (4.6%); TPC-borderline = 75/460 (16.3%); TPC-health = 81/460 (17.6%). **b** CCC. Sample size = 231. Rho = − 0.508; 95% confidence interval for Rho = − 0.598 to − 0.406. CCC-CF = 26/231 (11.26%); CCC-borderline = 108/231 (46.75%); CCC-health = 97/231 (41.99%). *p*-value < 0.0001. **c** SPC. Sample size = 52. Rho = − 0.361; 95% confidence interval for Rho = − 0.577 to − 0.098. SPC-CF = 5/52 (9.62%); SPC-borderline = 19/52 (36.54%); SPC-health = 28/52 (53.85%). *p*-value = 0.0086. **d** TPC. Sample size = 177. Rho = − 0.470; 95% confidence interval for Rho = − 0.577 to − 0.346. TPC-CF = 21/177 (11.86%); TPC-borderline = 75/177 (42.76%); TPC-health = 81/177 (45.76%). *p*-value < 0.0001. CCC, continuous constant current; SPC, sinusoidal pulsed current; TPC, triangular pulsed current; CF, cystic fibrosis. Chloride values (mmol/L): (i) CF – ≥ 60 mmol/L; (ii) borderline – between ≥ 30 to < 60 mmol/L; (iii) health – > 30 mmol/L. The chloride concentration is shown as milliequivalent by liter (mmol/L). Also, the sweat weight is shown as milligrams

**Fig. 7:**
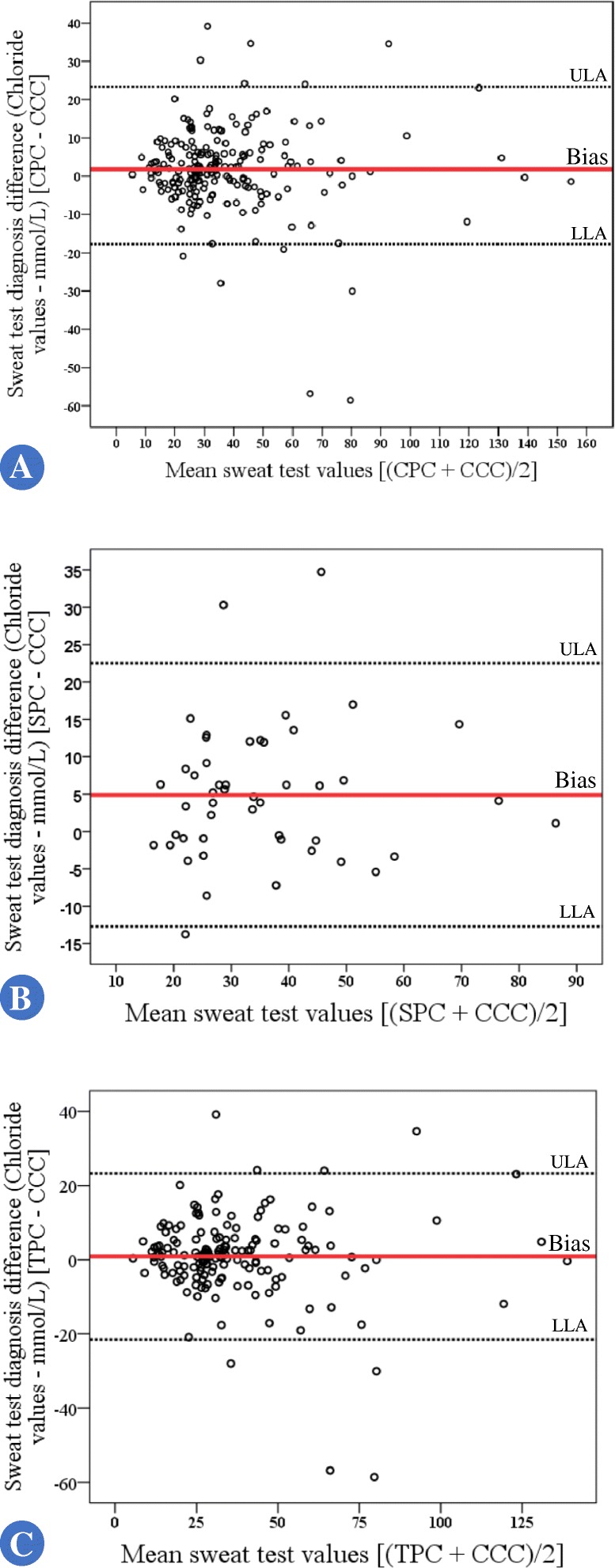
Bland and Altman plot considering the agreement between the sweat chloride values achieved from continuous pulsed current and continuous constant current. **a** Sweat chloride values achieved from continuous pulsed current and continuous constant current – *R* = 0.062; *R*^2^ = 0.004; *p*-value = 0.368; Bias = 1.806; ULA = 23.386; LLA = − 19.774. **b** Sweat chloride values achieved from sinusoidal pulsed current and continuous constant current – *R* = 0.027; *R*^2^ = 0.001; *p*-value = 0.855; Bias = 4.891; ULA = 22.502; LLA = − 12.72. **c** Sweat chloride values achieved from triangular pulsed current and continuous constant current – *R* = 0.074; *R*^2^ = 0.005; *p*-value = 0.349; Bias = 0.879; ULA = 23.254; LLA = − 21.496. ULA, upper limit of agreement; LLA, lower limit of agreement. The chloride concentration is shown as milliequivalent by liter (mmol/L). The statistical analysis was done with simple linear regression. Alpha = 0.05

No association was observed between sweat weight when compared with different currents (*p*-value > 0.05) (Table 2). However, the sweat test outcome was different according to the current applied and sweat weight achieved. Data are presented in Tables 3, 4, 5 and 6, which includes the Kappa agreement index calculation. Also, when we compared the triangular pulsed current and sinusoidal pulsed current regarding the difference between continuous constant current and continuous pulsed current, and the sinusoidal pulsed current showed a higher amount of sweat weight (*p*-value = 0.02) (Fig. 5). Moreover, there was a correlation (Spearman’s Rho) between sweat chloride values (CF-diagnosis) and the sweat weight of − 0.482 (95% confidence interval for Rho = − 0.549 to − 0.409) (*p*-value < 0.0001) (Fig. 6a-d). In addition, we included the Fig. 7 showing the results from Bland and Altman plot considering the agreement between the sweat chloride values achieved from continuous pulsed current (Fig. 7a) [sinusoidal pulsed current (Fig. 7b) and triangular pulsed current (Fig. 7c)] and continuous constant current. In the data, we found there is no proportional bias in our data and mean values are unrelated and only explain less than 8% of the variation.

**Table 2:** Comparison of number of exams with insufficient sweat weight (below 75 mg) between continuous constant current and sinusoidal and triangular continuous pulsed current

		Continuous constant current		
	Sweat weight	Sufficient	Insufficient	*p*-value
Sinusoidal pulsed current	Sufficient	49 (89.1%)	3 (5.5%)	0.655^a^
	Insufficient	2 (3.6%)	1 (1.8%)	
Triangular pulsed current	Sufficient	172 (83.9%)	9 (4.4%)	0.523^b^
	Insufficient	13 (6.3%)	11 (5.4%)	
Continuous pulsed current	Sufficient	221 (85%)	12 (4.6%)	0.333^c^
	Insufficient	15 (5.8%)	12 (4.6%)	

Statistical analysis conducted through McNemar’s tests. Alpha = 0.05

^a^Number of observed agreements = 50 (90.91%); Number of agreements expected by chance = 48.4 (88.07%); Kappa = 0.238; SE of kappa = 0.232; 95% confidence interval (95%CI) = − 0.217 to 0.693; strength of agreement = fair

^b^Number of observed agreements = 183 (93.85%); number of agreements expected by chance = 163.9 (84.04%); Kappa = 0.614; SE of kappa = 0.101; 95%CI = 0.416 to 0.813; strength of agreement = good

^c^Number of observed agreements = 233 (89.62%); number of agreements expected by chance = 214 (82.3%); Kappa = 0.413; SE of kappa = 0.093; 95%CI = 0.232 to 0.595; strength of agreement = moderate. As shown in the table, the minor prevalence of insufficient sweat weight occurred for sinusoidal pulsed current (1.8%)

**Table 3:** Comparison of sweat test result between continuous constant current and sinusoidal and triangular continuous pulsed current

Sweat test result		Continuous constant current			
		Cystic fibrosis	Borderline	Normal	*p*-value
Sinusoidal pulsed current	Cystic fibrosis	4	1	0	**0.008** ^**a**^
	Borderline	0	16	1	
	Normal	1	11	15	
Triangular pulsed current	Cystic fibrosis	14	5	0	0.423^b^
	Borderline	6	49	12	
	Normal	0	16	62	
Continuous pulsed current	Cystic fibrosis	18	6	0	**0.033** ^**c**^
	Borderline	6	65	13	
	Normal	1	27	77	

Statistical analysis conducted through Wilcoxon signed-rank test (ordinal data). Alpha = 0.05

^a^Number of observed agreements = 35 (71.43%); number of agreements expected by chance = 19 (38.86%); Kappa = 0.533; SE of kappa = 0.101; 95% confidence interval (95%CI) = 0.335 to 0.73; strength of agreement = moderate; weighted Kappa = 0.563; strength of agreement = moderate

^b^Number of observed agreements = 125 (76.22%); number of agreements expected by chance = 66.1 (40.31%); Kappa = 0.602; SE of kappa = 0.056; 95%CI = 0.492 to 0.711; strength of agreement = good; weighted Kappa = 0.664; strength of agreement = good

^c^Number of observed agreements = 160 (75.12%); number of agreements expected by chance = 85.8 (40.3%); Kappa = 0.583; SE of kappa = 0.05; 95%CI = 0.485 to 0.681; strength of agreement = moderate; weighted Kappa = 0.639; strength of agreement = good. Statistically significant data are in bold. Additional analysis is shown as Fig. 7

**Table 4:**
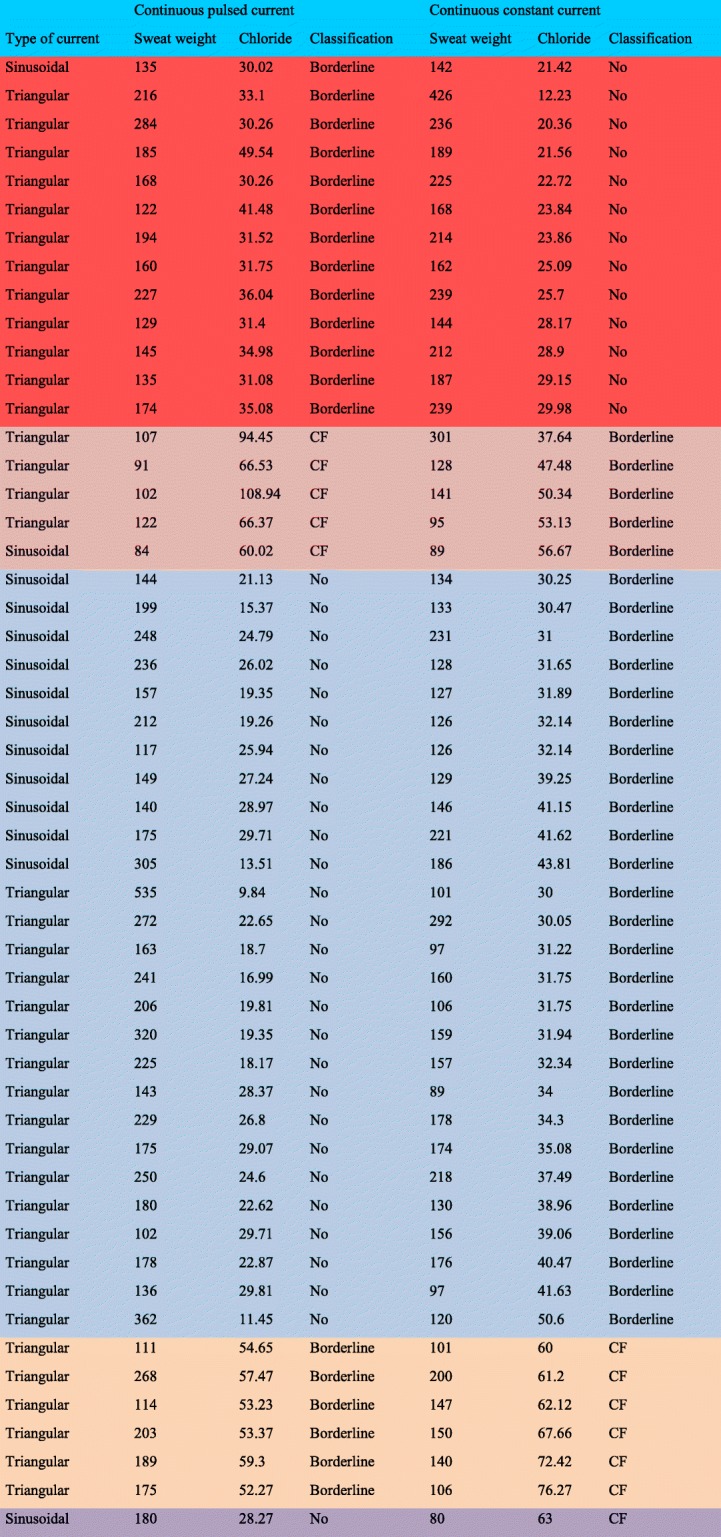
Individuals included in the study who presented different results in the cystic fibrosis (CF) classification according to the chloride dose

The colors indicate the changes in the cystic fibrosis diagnosis classification, according to the chloride dose. The table also shows the obtained sweat weight value, which is associated with the chloride dilution in the amount of sweat collected and may change the sweat test result. The sweat test is defined in mmol/L. The sweat weight is defined in mg. Using different currents, we achieved different results in the classification of the sweat test, mainly, considering the borderline value. Also, in the present study, we included only individuals with clinical suspicion of cystic fibrosis, but sometimes, without a close diagnosis (absence of two *CFTR* mutations and/or two sweat tests ≥ 60 mmol/L). In this context, we found a lot of variability that could be a reflex of the patient enrolled in the studied sample. Finally, all the currents used to induce sweating showed a close capacity to induce the sweat weight above 75 mg

**Table 5:** Comparison of sweat weight values (mg) between pulsed current and continuous current, considering the sweat test classification according to the chloride dose

Continuous pulsed current		Continuous constant current		N	*p*-value
Non-cystic fibrosis	108	Cystic fibrosis	80	1	–
Borderline	175 ± 46	Non-cystic fibrosis	214 ± 72	13	**0.009**
	168 (122 to 284)		212 (142 to 426)		
Cystic fibrosis	101 ± 15	Borderline	151 ± 87	5	0.138
	102 (84 to 122)		128 (89 to 301)		
Non-cystic fibrosis	215 ± 90	Borderline	154 ± 46	27	**< 0.001**
	199 (102 to 535)		146 (89 to 292)		
Borderline	181 ± 62	Cystic fibrosis	141 ± 36	6	0.075
	182 (111 to 269)		143 (101 to 20)		

Statistical analysis conducted through Wilcoxon signed-rank test. Statistically significant data are in bold. N, number of individuals included in each category – It does not reflect the number of individuals included in the performed experiments. Data are presented in table as: number of individuals; mean ± standard deviation; median (minimum to maximum), except for the first line where the absolute value is shown

**Table 6:** Comparison of sweat weight values (mg) between pulsed current and continuous current, considering if an increase or reduction was observed in chloride concentration between pulsed current and continuous current

	Continuous pulsed current	Continuous constant current	N	*p*-value
Reduction of chloride concentration was observed from continuous pulsed current to continuous constant current^a^	154 ± 52	197 ± 80	18	**0.004**
	140 (84 to 284)	0.188 (89 to 426)		
Increase of chloride concentration was observed from continuous pulsed current to continuous constant current^b^	207 ± 85	149 ± 45	34	**< 0.001**
	184 (102 to 535)	143 (80 to 292)		

Statistical analysis conducted through Wilcoxon signed-rank test. Statistically significant data are in bold

^a^data presented in Table 4 in orange and red

^b^data presented in Table 4 in blue, green, and yellow. The obtained sweat weight was associated with chloride concentration and may change the sweat test result because the amount of sweat collected could be cause in a dilution of the chloride in the own sweat. Statistically significant data are in bold. Data are presented in table as: number of individuals; mean ± standard deviation; median (minimum to maximum)

## Discussion

Our findings show that the type of current used to induce sweat can alter the electrical impedance created among the electrode, gauze and skin components. This fact influences the induction of sweat and, consequently, the sweat weight, promoting a variability at the sweat test results. In addition, gender and ethnicity may influence the natural variation of sweat test values and should be considered when conducting the test [25–27].

Few studies are available taking into account the different types of currents to promote the skin sweat induction in CF. In a previous study from our group we observed that the first stage of the sweat test (sweat induction) presents particularities that need a better investigation and technique detailing [23].

The sweat test has been considered, since the 1950s, as the gold standard for the diagnosis of CF [1, 28, 29]. However, still today, numerous studies have shown that this test involves challenges, requiring sweat test standardization [2, 11–13, 30–33]. Like other countries, Brazil, including in CF reference centers, presents low knowledge about how to conduct the sweat test protocol and the accepted methods to perform chloride dosage [20].

To facilitate the sweat test conduction in Brazil, our research group recently conducted a study in which the sweat induction was evaluated through pilocarpine iontophoresis using a device developed by the biomedical engineering team of the university. In the study, we evaluated: (i) the outcomes from the use of continuous constant current and triangular pulsed current; (ii) amount of sweat induced by different currents; (iii) ideal time of sweat induction and collection; (iv) electrode, gauze and skin electrical impedance for the different currents; (v) side effects. Hence, a better performance of the sweat test was obtained with one mA current, 1000 Hz frequency (for triangular pulsed current), 10 and 30 min for induction and collection, respectively. In addition, no side effects were observed that would make the developed device unfeasible [23].

Based on the previous findings, in this study, we proposed to evaluate a larger sample of participants and to analyze sweat induction at different ages, and in different genders and ethnic groups, comparing the sinusoidal pulsed current, triangular pulsed current and continuous constant current. At the sweat test, the amount of sweat produced is directly related to the delivery of pilocarpine in the skin, and when the induction by iontophoresis is incorrectly performed, the induced sweat may be insufficient and can alter the final result of the diagnosis. Aggravation and risks arising from the electric currents can be observed due to errors during sweat induction, with the possibility of burns, especially in newborns.

Our study showed that the electrode, gauze and skin assembly electrical impedance, providing a larger or smaller amount of sweat, in an inverse association with sweat weight, varied according to the type of current applied. The sinusoidal pulsed current resulted in lower electrical impedance and higher sweat production, when compared with continuous constant current and triangular pulsed current. However, although the sinusoidal pulsed current resulted in lower electrical impedance and higher sweat production, all currents evaluated were able to induce sufficient sweat for the electrolyte analysis. In addition, in our sample, electrical impedance showed a positive correlation with age with all types of currents applied (continuous constant current – Spearman’s Rho = 0.262; *p*-value < 0.001; triangular pulsed current – Spearman’s Rho = 0.256, *p*-value < 0.001; sinusoidal pulsed current – Spearman’s Rho = 0.292, *p*-value = 0.032).

In the evaluation of iontophoresis for the delivery of drugs to the skin, continuous constant current was the most frequent current [34]. However, according to other authors, the use of continuous constant current may result in permanent electrode, gauze and skin assembly polarization during sweat induction and reduce the efficiency of iontophoretic administration in proportion to the current application time, by increasing the impedance of the electrode-skin assembly, and this may cause burning and redness [35–37].

In contrast, some authors have shown that continuous pulsed current can minimize the presence of polarization [37, 38]. To prevent the side effects of continuous constant current, some researchers have studied several types of drugs and evaluated the efficacy of continuous pulsed current, at different waveforms, in skin permeation. However, no consensus has been achieved regarding the most effective type of current.

What is known so far is that for continuous pulsed current, the waveform influences the permeation of drugs through the skin. For instance, (a) the absorption of luteinizing hormone-releasing hormone using continuous constant current (0.764 mA/cm^2^) and sinusoidal and rectangular continuous pulsed current (0.764 mA/cm^2^ and one kHz) did not produce different values for the permeation flux. However, the flow caused by triangular waveforms was lower than that of continuous constant current [39]; (b) the permeation flow of some drugs is efficient with the use of sinusoidal, trapezoidal and rectangular waveforms (0.33 mA/cm^2^ and two kHz) [40]; (c) the skin permeability of amino acids (lysine and glutamic acid) using current density of 0.5 mA/cm^2^ and 2.5 kHz frequency were the same in rectangular and sinusoidal waveforms [41]; (d) the permeability of iondomethacin was better with continuous pulsed current at frequencies lower than 100 Hz and with rectangular and sinusoidal waveforms [42].

On the other hand, square waveform was more efficient in promoting the permeation of the granisetron by iontophoresis than the continuous constant current [37]. The higher efficiency of square pulsed current in relation to continuous constant current can be explained by the amount of electrical permeation load that is reduced by half with square continuous pulsed current. Square continuous pulsed current was also considered less harmful to the skin.

Unlike previous studies, our study used triangular pulsed current, sinusoidal pulsed current, and a fixed value of one mA. Although the sinusoidal pulsed current presented lower electrical impedance when compared with the triangular pulsed current and continuous constant current, all tested currents were able to provide sufficient sweat weight for the electrolyte analysis.

In our previous study, the triangular pulsed current presented lower electrical impedance values when compared with continuous constant current, but without difference in sweat weight [23]. On the other hand, in this study, we identified that the sinusoidal pulsed current presented the lowest electrical impedance, which was concomitant to the greatest sweat weight obtained. The results of our two studies (the one of 2014 and this study) favor the use of continuous pulsed current to obtain sweat for the sweat test, and that the sinusoidal pulsed current is probably more efficient than the triangular pulsed current.

Further studies on sweat induction with iontophoresis and pilocarpine using continuous pulsed current should be conducted to obtain more consistent information on sweat induction and the use of different types of currents.

The literature describes that skin characteristics change with age, and that such changes have an impact on skin impedance and resistance, making it difficult to deliver medication during electrical stimulation. With aging, the skin tends to become dehydrated, dry, and more resistant, which are obstacles to sweat induction during stimulation [43–45]. These factors may explain the positive correlation between electrical impedance and age observed in our sample, regardless of the current applied.

In our study, the age of the participants did not influence the collection of sufficient or insufficient amount of sweat to measure chloride at the sweat test (*p*-value for continuous constant current, sinusoidal pulsed current and triangular pulsed current were 0.098, 0.661, and 0.468, respectively).

Also known is the fact that high body mass index can hinder drug permeation by iontophoresis, and it may also change the pilocarpine permeation in the skin and then reduce sweat production. This fact may be associated with the characteristic poor electrical conduction of adipose tissues [21, 42, 46]. However, in our study, most participants did not present high body mass index, which could change the results obtained. Higher body mass index values were identified in Caucasians when compared with non-Caucasians.

It is also known that the stratum corneum is the outermost layer of the skin and, besides the presence of lipids, keratin restricts the transport of compounds through the skin, for example, pilocarpine [43, 47, 48]. The literature reports that increased keratin concentration of the skin is associated with difficult sweat induction [49–51]. However, in our study, electrical impedance was observed in Caucasians only with sinusoidal pulsed current application.

In our study, the male participants presented higher amounts of sweat with all tested currents, and lower values of electrical impedance with continuous constant current and continuous pulsed current. This fact may be associated with greater sudomotor activity in males [44, 52] and due to the skin structure. Our findings agree with the evidence found in the literature showing that males present greater sweat weight [53, 54].

A limiting factor for the discussion of our findings is the lack of data in the literature related to the sweat induction stage of the sweat test involving pilocarpine iontophoresis and the use of different types of currents. But, using different currents, we achieved different results in the classification of the sweat test, mainly, considering the borderline value. Also, in the present study, we included many individuals with clinical suspicion of CF, but sometimes, without a close diagnosis (absence of two *CFTR* mutations and/or two sweat tests ≥ 60 mmol/L). In this context, we found a lot of variability that could be a singular characteristic of the patient enrolled in the studied sample. And, in our work, all the currents showed a close capacity to induce the sweating.

Regardless of the current applied, the guidelines must be strictly followed when conducting the sweat test. In the method of Gibson and Cooke, some additional precautions should be taken, such as the use of gauze completely soaked with pilocarpine and of a proper size to protect the skin from the contact with the electrode, the electrode size should be standardized, since the current density should not exceed 0.16 mA/cm^2^; the distance between the electrodes should be enough to induce the permeation of pilocarpine, and in our study, the minimum distance of two cm and maximum distance of five cm in the forearm were considered ideal for the results obtained.

Our study points to the need to properly assess the sweat weight. In our data, a large number of the evaluated individuals had a change in the outcome of the CF diagnosis, considering the increase in sweat weight collected in the sweat test. At the same time, we showed that there is a negative correlation between the chloride ion and the sweat weight, and this fact was observed in the literature [26], but other studies should be performed to close the idea behind this issue. Thus, the sweat test should be redone even when a sweat value was obtained according to the guidelines (≥ 75 mg) and there was still clinical suspicion and/or positive neonatal screening. In cases where the suspicion remains after many sweat tests performed, the genetic test is necessary to confirm or not the CF diagnosis.

In the literature, we did not have a consensus regarding the maximum value to sweat weight where we can consider the sweat test as acceptable to CF diagnosis. Also, as previously reported by us and discussed in our present data, we found a negative correlation between sweat weight and concentration of chloride [26]. In this scenario, we believe that this fact is not important when the final sweat weight is bigger than 75 mg until values around 100 mg, but when we have a highest sweat weight, a major influence in concentration of chloride can be achieved and representing an alteration in the classification of the patient, mainly, in cases, with normal or borderline values in sweat test.

Additional studies on sweat test should be conducted with sweat samples obtained from healthy individuals and individuals with CF using other types of waveform, different body mass indexes and ethnic groups as well as comparisons between different age groups. In addition, in our study design, we did not follow strictly the guidelines [6, 8, 15, 16, 24] i.e. we used 10 min (versus five min in the guidelines) and one mA (versus 2.5 to four mA in the guidelines) to perform the sweat induction. Moreover, with the lower mA, we needed to include a greater lapse of time to promote the sweat induction.

## Conclusion

The evaluated currents are able to induce and produce sweat in sufficient amounts for chloride analysis. Moreover, the sinusoidal pulsed current presented lower electrical impedance when compared with the other currents and we suggest its use to induce sweat in patients with sweat problems. Also, ethnicity, gender, age and body mass index did not influence sweat induction at the sweat test, and no side effect was observed in our study.

## Additional files

Additional file 1: Comparison of continuous constant current, sinusoidal pulsed current and triangular pulsed current for impedance and sweat weight. The sinusoidal pulsed current showed a minor impedance when compared with triangular pulsed current and continuous constant current. Also, the sinusoidal pulsed current was able to induce a better sweat weight than continuous constant current. (DOCX 21 kb)

Additional file 2: Gender comparison for the sweat test markers evaluated in our study. In all types of currents in use, females showed a low amount of sweat weight. Moreover, females have a higher impedance in use of triangular pulsed current and continuous constant current than males. (DOCX 20 kb)

Additional file 3: Comparison of ethnicity for the sweat test markers evaluated in our study. Caucasians have a higher body mass index than no Caucasians in the triangular pulsed current and continuous constant current groups. Also, Caucasians showed a lower Impedance than no Caucasian to sinusoidal pulsed current. (DOCX 20 kb)

## Abbreviations

BMI: Body mass index
CCC: Continuous constant current
CF: Cystic fibrosis
CFTR: Cystic fibrosis transmembrane regulator
CPC: Continuous pulsed current
IRMS: Effective current measure (current root mean square)
OMIM: Online Mendelian Inheritance in Man
RMS: Root mean square
SPC: Sinusoidal pulsed current
ST: Sweat test
TPC: Triangular pulsed current
VRMS: Effective voltage measure (volts root mean square)
Z: Composite impedance
