# Supplementary material for: Evaluation of continuous constant current and continuous pulsed current in sweat induction for cystic fibrosis diagnosis
Source: BMC Pulm Med. 2018 Sep 14;18:153. doi: 10.1186/s12890-018-0696-3 (PMC6137935; doi:10.1186/s12890-018-0696-3)
Supplement: Supplementary file 1 — Comparison of continuous constant current, sinusoidal pulsed current and triangular pulsed current for impedance and sweat weight. The sinusoidal pulsed current showed a minor impedance when compared with triangular pulsed current and continuous constant current. Also, the sinusoidal pulsed current was able to induce a better sweat weight than continuous constant current. (DOCX 21 kb) [file 12890_2018_696_MOESM1_ESM.docx]

**Title:** Evaluation of continuous constant current and continuous pulsed current in sweat induction for cystic fibrosis diagnosis

**Additional file 1.** Comparison of continuous constant current, sinusoidal pulsed current and triangular pulsed current for impedance and sweat weight. Only the data with significant p-values are shown.

|  | **Type of current** | | | | |  |
| --- | --- | --- | --- | --- | --- | --- |
|  | **Sinusoidal** | **Triangular** | | | | **p-value** |
| **Impedance (Ω)** | 54; 6.14±2.08; 6.23  (2.12 to 11.05); 5.57 to 6.71 | 201; 7.94±3.16; 7.82  (1.94 to 17.76); 7.5 to 8.38 | | | | < 0.001 |
| **Marker** | **Sinusoidal** | | | **Continuous** | | **p-value** |
| **Impedance (Ω)** | 54; 6.14±2.08; 6.23  (2.12 to 11.05); 5.57 to 6.71 | | | 54; 7.29±2.98; 6.76  (2.95 to 17.11); 6.47 to 8.1 | | < 0.001 |
| **Sweat weight (mg)** | 55; 178±70; 175  (46 to 433); 159 to 197 | | 55; 146±46; 140  (50 to 234); 133 to 158 | | | < 0.001 |
| **Marker** | **Triangular** | | | | **Continuous** | **p-value** |
| **Impedance (Ω)** | 197; 7.97±3.2; 7.83  (1.94 to 17.76); 7.52 to 8.41 | | | | 197; 8.9±4.8; 8.15  (1.12 to 38.33); 8.23 to 9.6 | < 0.001 |

Data are presented as: number of individuals; mean±standard deviation; median (minimum to maximum); confidence interval for the mean value. Statistical analysis conducted through Wilcoxon signed-rank test and Mann-Whitney U test of independent samples. Alpha = 0.05. The currents are shown as Ω using the following equation: [Z = V_RMS_ / I_RMS_ (Ω)]; Z = composite impedance (Ω); V_RMS_ = effective voltage measured; I_RMS_ = effective current measured. Also, the sweat weight is shown as milligrams.
