# Supplementary material for: Evaluation of continuous constant current and continuous pulsed current in sweat induction for cystic fibrosis diagnosis
Source: BMC Pulm Med. 2018 Sep 14;18:153. doi: 10.1186/s12890-018-0696-3 (PMC6137935; doi:10.1186/s12890-018-0696-3)
Supplement: Supplementary file 2 — Gender comparison for the sweat test markers evaluated in our study. In all types of currents in use, females showed a low amount of sweat weight. Moreover, females have a higher impedance in use of triangular pulsed current and continuous constant current than males. (DOCX 20 kb) [file 12890_2018_696_MOESM2_ESM.docx]

**Title:** Evaluation of continuous constant current and continuous pulsed current in sweat induction for cystic fibrosis diagnosis

**Additional file 2.** Gender comparison for the sweat test markers evaluated in our study. Only the data with significant p-values are shown.

| **Continuous constant current** | | | | | |
| --- | --- | --- | --- | --- | --- |
| **Marker** | **Female** | | **Male** | **p-value** | |
| **Impedance (Ω)** | 136; 9.3±4.94; 7.91  (1.12 to 38.33); 8.46 to 10.14 | | 116; 7.67±3.75; 7.53  (2.30 to 22.29); 6.98 to 8.36 | 0.008 | |
| **Marker** | **Female** | | **Male** | **p-value** | |
| **Sweat weight (mg)** | 141; 155±73; 146  (0 to 425); 142 to 167 | | 119; 193±104; 186  (3 to 577); 174 to 211 | < 0.001 | |
| **Sinusoidal pulsed current** | | | | | |
| **Marker** | **Female** | | **Male** | | **p-value** |
| **Sweat weight (mg)** | 30; 153±55; 158  (46 to 267); 132 to 174 | | 25; 208±0.074; 209  (97 to 433); 177 to 238 | | 0.007 |
| **Triangular pulsed current** | | | | | |
| **Marker** | | **Female** | **Male** | **p-value** | |
| **Impedance (Ω)** | | 109; 8.57±3.17; 8.16  (2.33 to 17.76); 7.97 to 9.17 | 92; 7.2±2.98; 7.18  (1.94 to 16.33); 6.58 to 7.81 | 0.003 | |
|  | **Female** | | **Male** | **p-value** | |
| **Sweat weight (mg)** | 109; 164±88; 157  (6 to 535); 148 to 181 | | 92; 206±115; 189  (9 to 699); 182 to 229 | 0.003 | |

Data are presented as: number of individuals; mean±standard deviation; median (minimum to maximum); confidence interval for the mean value. Statistical analysis conducted through Mann-Whitney U test of independent samples. Alpha = 0.05. The currents are shown as Ω using the following equation: [Z = V_RMS_ / I_RMS_ (Ω)]; Z = composite impedance (Ω); V_RMS_ = effective voltage measured; I_RMS_ = effective current measured. Also, the sweat weight is shown as milligrams.
