# Supplementary material for: Evaluation of continuous constant current and continuous pulsed current in sweat induction for cystic fibrosis diagnosis
Source: BMC Pulm Med. 2018 Sep 14;18:153. doi: 10.1186/s12890-018-0696-3 (PMC6137935; doi:10.1186/s12890-018-0696-3)
Supplement: Supplementary file 3 — Comparison of ethnicity for the sweat test markers evaluated in our study. Caucasians have a higher body mass index than no Caucasians in the triangular pulsed current and continuous constant current groups. Also, Caucasians showed a lower Impedance than no Caucasian to sinusoidal pulsed current. (DOCX 20 kb) [file 12890_2018_696_MOESM3_ESM.docx]

**Title:** Evaluation of continuous constant current and continuous pulsed current in sweat induction for cystic fibrosis diagnosis

**Additional file 3.** Comparison of ethnicity for the sweat test markers evaluated in our study. Only the data with significant p-values are shown.

| **Continuous constant current** | | | | |
| --- | --- | --- | --- | --- |
| **Marker** | | **No Caucasian** | **Caucasian** | **p-value** |
| **Body mass index (Kg/m^2^)** | | 125; 21.26±5.7; 20.21  (11.06 to 42.29); 20.25 to 22.27 | 133; 23.5±6.07; 23.72  (0 to 38.2); 22.46 to 24.55 | 0.001 |
| **Sinusoidal pulsed current** | | | | |
| **Marker** | **No Caucasian** | | **Caucasian** | **p-value** |
| **Impedance (Ω)** | 20; 6.98±2.36; 7.55  (3.19 to 11.05); 5.87 to 8.09 | | 34; 5.64±1.74; 5.66  (2.12 to 9.62); 5.03 to 6.25 | 0.037 |
| **Triangular pulsed current** | | | | |
| **Marker** | **No Caucasian** | | **Caucasian** | **p-value** |
| **Body mass index (Kg/m^2^)** | 105; 20.6±5.06; 19.57  (11.06 to 34.84); 19.61 to 21.58 | | 98; 23.14±6.19; 22.9  (0 to 38.2); 21.89 to 24.38 | 0.001 |

Data are presented as: number of individuals; mean±standard deviation; median (minimum to maximum); confidence interval for the mean value. Statistical analysis conducted through Mann-Whitney U test of independent samples. Alpha = 0.05. The currents are shown as Ω using the following equation: [Z = V_RMS_ / I_RMS_ (Ω)]; Z = composite impedance (Ω); V_RMS_ = effective voltage measured; I_RMS_ = effective current measured.
